# Development of Biopolymeric Hybrid Scaffold-Based on AAc/GO/nHAp/TiO_2_ Nanocomposite for Bone Tissue Engineering: In-Vitro Analysis

**DOI:** 10.3390/nano11051319

**Published:** 2021-05-17

**Authors:** Muhammad Umar Aslam Khan, Wafa Shamsan Al-Arjan, Mona Saad Binkadem, Hassan Mehboob, Adnan Haider, Mohsin Ali Raza, Saiful Izwan Abd Razak, Anwarul Hasan, Rashid Amin

**Affiliations:** 1 BioInspired Device and Tissue Engineering Research Group, School of Biomedical Engineering and Health Sciences, Faculty of Engineering, Universiti Teknologi Malaysia, Skudai 81300, Johor, Malaysia; saifulizwan@utm.my; 2 Department of Polymer Engineering and Technology, University of the Punjab, Quaid-e-Azam Campus, Lahore 54590, Pakistan; 3 Institute for Personalized Medicine, School of Biomedical Engineering, Shanghai Jiao Tong University, Shanghai 200030, China; 4 Department of Metallurgy and Materials Engineering, CEET, University of the Punjab, Quaid-e-Azam Campus, Lahore 54590, Pakistan; mohsin.ceet@pu.edu.pk; 5 Department of Chemistry, College of Science, King Faisal University, Al-Ahsa 31982, Saudi Arabia; walarjan@kfu.edu.sa; 6 Department of Chemistry, Faculty of Science, University of Jeddah, Jeddah 21589, Saudi Arabia; mbinkadem@uj.edu.sa; 7 Department of Engineering Management, College of Engineering, Prince Sultan University, Rafha Street, Riyadh 11586, Saudi Arabia; hmehboob@psu.edu.sa; 8 Department of Biological Sciences, National University of Medical Sciences, Rawalpindi, Punjab 46000, Pakistan; adnan_phd@outlook.com; 9 Center for Advanced Composite Materials, Universiti Teknologi Malaysia, Skudai 81300, Johor, Malaysia; 10 Biomedical Research Center, Qatar University, Doha 2713, Qatar; hasan.anwarul.mit@gmail.com; 11 Department of Mechanical and Industrial Engineering, Qatar University, Doha 2713, Qatar; 12 Department of Biology, College of Sciences, University of Hafr Al Batin, Hafr Al Batin 39524, Saudi Arabia; rashida@uhb.edu.sa

**Keywords:** biocompatibility, biodegradation, cytotoxicity, drug delivery, graphene oxide, hybrid scaffolds, nanocomposite, tissue engineering

## Abstract

Bone tissue engineering is an advanced field for treatment of fractured bones to restore/regulate biological functions. Biopolymeric/bioceramic-based hybrid nanocomposite scaffolds are potential biomaterials for bone tissue because of biodegradable and biocompatible characteristics. We report synthesis of nanocomposite based on acrylic acid (AAc)/guar gum (GG), nano-hydroxyapatite (HAp NPs), titanium nanoparticles (TiO_2_ NPs), and optimum graphene oxide (GO) amount via free radical polymerization method. Porous scaffolds were fabricated through freeze-drying technique and coated with silver sulphadiazine. Different techniques were used to investigate functional group, crystal structural properties, morphology/elemental properties, porosity, and mechanical properties of fabricated scaffolds. Results show that increasing amount of TiO_2_ in combination with optimized GO has improved physicochemical and microstructural properties, mechanical properties (compressive strength (2.96 to 13.31 MPa) and Young’s modulus (39.56 to 300.81 MPa)), and porous properties (pore size (256.11 to 107.42 μm) and porosity (79.97 to 44.32%)). After 150 min, silver sulfadiazine release was found to be ~94.1%. In vitro assay of scaffolds also exhibited promising results against mouse pre-osteoblast (*MC3T3-E1*) cell lines. Hence, these fabricated scaffolds would be potential biomaterials for bone tissue engineering in biomedical engineering.

## 1. Introduction

Bone is a porous natural composite with a variety of properties that can be used to heal or repair a fractured bone by replacing or reconstructing new tissues [1]. Artificial tissue grafting has become increasingly popular in recent years as a way to overcome the limitations of traditional approaches (i.e., allograft or autografts). Immune rejection, pathogen transfer, pain, infection, and limited availability are all addressed [2,3]. Tissue engineering (TE) is a cutting-edge method of constructing and designing scaffolds that combines materials engineering and life science techniques to aid in the reconstruction and regeneration of new tissues. Bone regeneration, based on scaffolds, is vital for healing bone defects caused by trauma, tumors, resection, and bone deformities. For successful defected bone regeneration, interaction between bone-related cells and scaffolds is critical [4].

The key benefit of TE is the ability to choose a cost-effective and optimized method for scaffold designing that closely resembles the fracture site. The synthesis of scaffolding composite materials for TE is still difficult due to the complex human body and sensitive biological system. Engineering bioresorbable, biodegradable, and biocompatible scaffolds for tissue regeneration is a feasible alternative to meet the standard requirement for host bone [5,6]. With controlled degradations, these fabricated scaffolds should not cause cytotoxic, immunogenic, inflammatory, or any other host reaction, allowing for new tissue regeneration [7,8]. The surface properties of the scaffolds (physicochemical and morphological) are essential to establish tissue response and cell growth. For bone tissue engineering, a scaffold with optimized pore size and interconnected porosity is desirable [9]. Scaffolds with the desired physical shape support vascularization of ingrown tissues. It is essential for cell penetration and migration to effectively vascularize the growth of new tissue to have maximum porosity (90%) and suitable pore diameter (minimum 100 and maximum 450 μm) [10,11]. For the cost-effective industrial development of bone tissue engineering scaffolds, economic synthesis methods are preferable.

Polymers (e.g., polysaccharides, poly(-hydroxy ester), hydrogels, or thermoplastic elastomers) have been used as biomaterials for TE in the previous era [12,13]. Different essential minerals or bioactive ceramics (such as calcium phosphates and bioactive glasses or glass ceramics) have been investigated to strengthen the microstructure that maintains structural integrity to grow new tissues. Recently, hybrid composites based on polymers and ceramics have received attention to achieve microstructural, physicochemical, and biological activities to enhance their interaction with host tissue [14]. Additionally, efforts were made to develop polymeric composite scaffolds with sustained delivery of bioactive molecules (including growth factors or antibiotics) to heal tissues or treat fractured bones to aid wound healing [11]. For bone regeneration, bioceramics such as hydroxyapatite (HAp), tricalcium phosphate (TCP), and bioactive glass (BGs) have been widely used. Due to their chemical similarity to the mineral phase of natural bone, these bioceramics are extremely biocompatible. The osteogenesis process also involves the interaction of osteoblast and osteoclast cells with bioceramics in order to repair or heal fractured bone [15]. The bioceramics stimulate osteoblast cells to differentiate and proliferate, which has a lot of potential in dental and orthopedic applications. These bioceramics have also been used to coat metallic implants in order to improve their interaction with host bone tissue, but they are brittle, resulting in poor mechanical strength. These bioactive ceramics are complicated to shape into a desired shape, like HAp, which has a long degradation time but low mechanical strength due to its brittle nature. Because of its favorable osteoconductivity, HAp, one of the substantial constituents of native bone, has been frequently used in bone tissue engineering [16,17]. In load-bearing applications, the porous structure of HAp cannot hold up. This critical issue can be carefully addressed by using polymeric composites to improve microstructural features. Bioceramics (i.e., HAp, BGs, and TCP) may have optimized microstructures for defected bone by combining biodegradable polymers (such as poly(glycolic acid), chitosan, arabinoxylan, and guar gum) with biodegradable polymers (including poly(glycolic acid), chitosan, arabinoxylan, and guar gum) [18]. These biodegradable polymers also act as a binder that reduces the brittleness of HAp. To achieve porous scaffolds, several methods have been previously reported to fabricate biopolymer composite scaffolds (often including solvent casting and particulate leaching or phase separation, etc.). The freeze-drying method, on the other hand, can produce more porosity with appropriate pore size. As a result, freeze-drying is a common method to fabricate porous scaffolds that can be controlled by ice crystal growth [19].

In this study, we developed polymeric nanocomposites with GG, acrylic acid (AAc), nano-hydroxyapatite (HAp NPs), nano titanium oxide (TiO_2_ NPs), GO, and N,N’-methylene bisacrylamide (NN-MBA). The homogenized slurry of these hybrid polymeric nanocomposites was poured into a 24-well cell culture plate, and the freeze-dry method was used to fabricate porous scaffold. FTIR, SEM/EDS, and UTM were used to characterize the structural, morphological, and mechanical properties of these hybrid nanocomposite scaffolds. Water contact angle, biodegradation (phosphate buffer saline (PBS) solution), and swelling analyses (water and PBS solution) were used to determine wetting, biodegradation, and swelling behavior of these nanocomposite scaffolds. A PBS solution was used to determine the drug release profile of these hybrid nanocomposite scaffolds. Brunauer–Emmett–Teller (BET) was used to determine pore size and porosity. These hybrid nanocomposite scaffolds were tested in vitro against *MC3T3-E1* cell lines. These hybrid nanocomposite scaffolds have the potential to be used as biomaterials in bone tissue engineering.

## 2. Materials and Methods

### 2.1. Materials

Guar gum is a natural polymer and is isolated from the seeds of *Cyamopsis tetragonoloba*. Its pH is 6.4, and it contains galactomannan polysaccharides. Sigma-Aldrich Malaysia (Selangor, Malaysia) supplied guar gum (CAS# G4129-500G), acrylic acid (AAc), and N,N’-methylene bisacrylamide (MBAA). Graphene oxide flake (CAS# 763713-1G) contains carbon (44.4%) and water (15.5%), and is amphiphilic in nature. It improves the physicochemical, mechanical, and biocompatibility properties of the polymeric nanocomposites materials. Hydroxyapatite nanoparticles (<100 nm particle size, ≥95%), titanium dioxide nanoparticles (<100 nm particle size, ≥98.3%), phosphate buffer saline (PBS) solution, and ethanol were supplied by Sigma-Aldrich, Selangor, Malaysia. All chemicals and reagents were analytically graded and used without any purification.

The *MC3T3-E1* cell lines were supplied by the American Type Culture Collection, Manassas, VA 20110, USA. L-glutamine penicillin/streptomycin, Alpha-MEM (*α-MEM*), and fetal bovine serum (*FBS*) were purchased from ThermoFisher Scientific (Waltham, MA, USA), Hyclone Laboratories Inc. (Logan, UT, USA).

### 2.2. Synthesis of Polymeric Nanocomposite

**Scheme 1:**
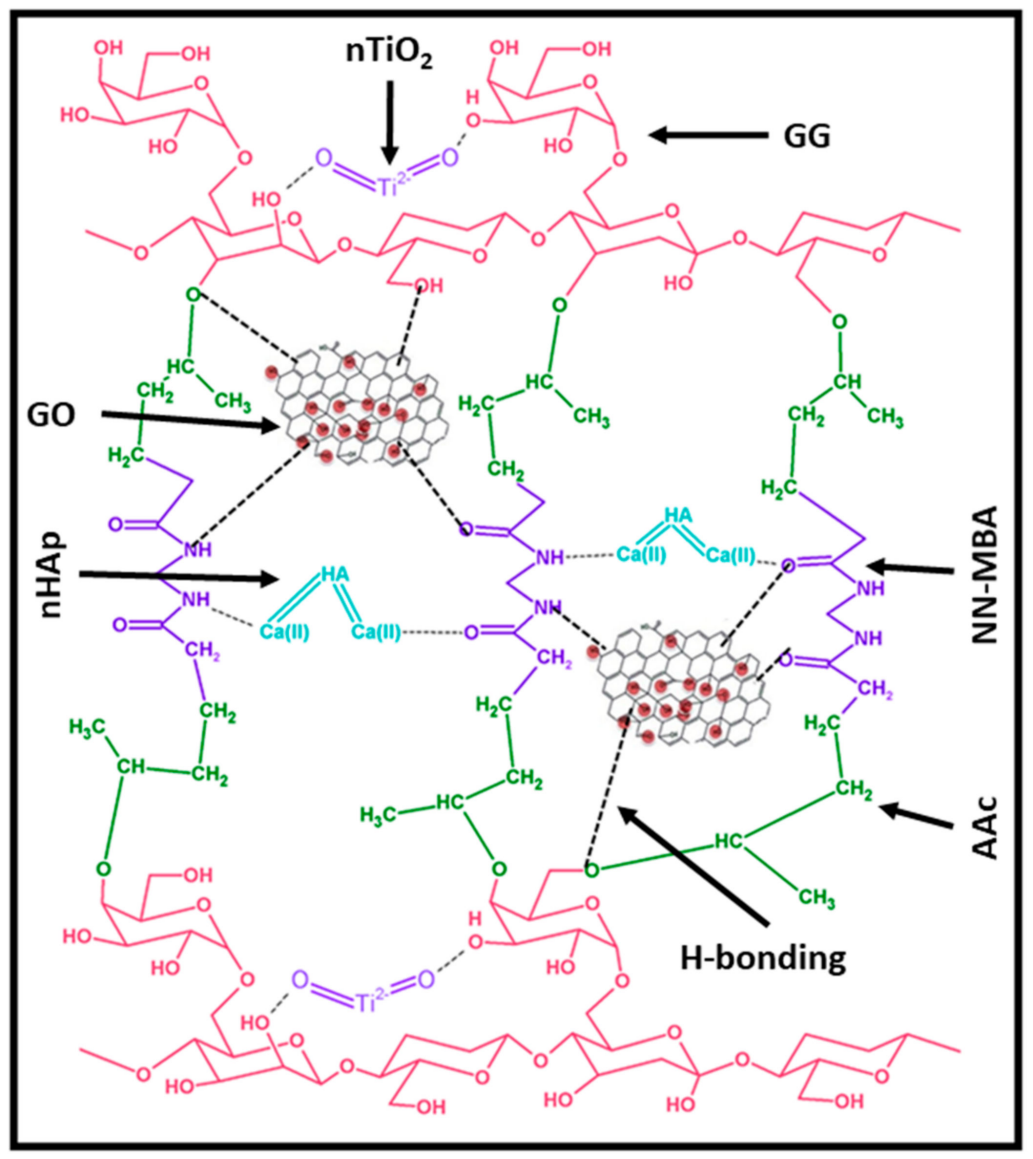
The proposed chemical schematic reaction mechanism presenting the synthesis of polymeric nanocomposite via free radical polymerization reaction and among the organic (GG, AAc, GO, and NN-MBA) and inorganic materials (nHAp and nTiO_2_).

**Figure 1:**
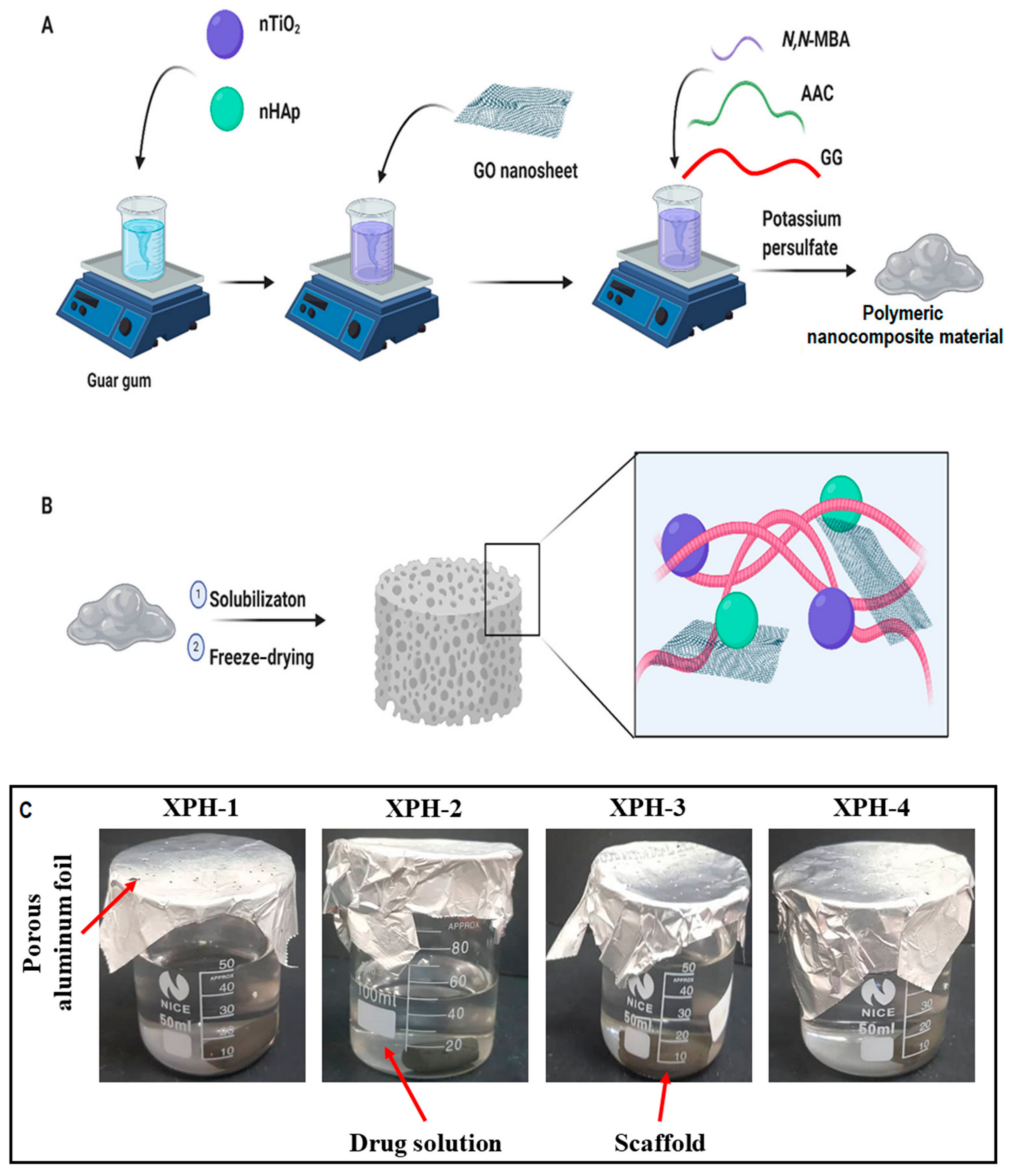
(**A**) Synthesis of polymeric matrix of nanocomposite through free radical polymerization. (**B**) Fabrication of porous scaffold via freeze-drying technique. (**C**) Silver sulfadiazine drug was loaded by a simple dip and dry method.

Guar gum powder (2 g) was dispersed in deionized water (30 mL), and the solution was transferred to a two-neck round bottom flask. HAp-NPs (1.4 g) and different quantities of TiO_2_ NPs (0.1, 0.2, 0.3, and 0.4 g) were suspended in the deionized water, and the suspension was added to the two-neck round bottom flask. The mixture was stirred for 45 min. After 45 min, GO (0.3 mg) was added to the mixture being optimized in our previous work [5]. The mixture was stirred to homogenize for 30 min at 65 °C under a nitrogen atmosphere. The homogenization of the mixture was followed by the addition of monomer (AAc (0.50 mL), crosslinker (MBA) crosslinker (0.05% of AAc), and initiator (potassium persulfate (0.05 g)). The reaction was carried out for 3 h at 65 °C under a nitrogen environment. After completing the reaction, heating was stopped, nitrogen gas was removed, reaction media were allowed to cool, and then the mixture was vacuum-filtered. The unreacted chemicals were removed from the product by washing the reaction media 3 to 4 times with excessive deionized water. The polymeric hybrid nanocomposite materials were oven-dried overnight at 50 °C. The proposed chemical schematic reaction mechanism is presented in the Scheme 1. The dried powder was saved in zip-lock plastic bags for further use and labeled (XPH-1, XPH-2, XPH-3, and XPH-4) corresponding to the different amounts of TiO_2_ NPs (0.1, 0.2, 0.3, and 0.4 g). The schematic of the proposed chemical is illustrated in Figure 1A.

### 2.3. Fabrication of Porous Hybrid Nanocomposite Scaffolds

The polymeric composite hybrid nanocomposite scaffolds were fabricated through the freeze-drying technique. Briefly, the polymeric hybrid nanocomposites powder (5 g) of the corresponding formulation was dispersed in deionized water to make the slurry and was poured into a cell culture plate (24-well) to freeze at −80 °C for 48 h. The dimension of each well of 24 cell culture plate is as follows: height = 1.74 cm, diameter = 1.56 cm. It was then freeze-dried to achieve porous, cylindrical, and well-dried scaffold without any crack or deformation as presented in Figure 1B.

### 2.4. Drug Loading

Silver sulfadiazine is a famous antibacterial drug, and it is helpful in killing disease or infection-causing pathogens during and after implantation. Silver sulfadiazine (2.5 mg) was weighed carefully and dissolved in ethanol (5 mL). The dissolved drug was then dropwise added in deionized water (45 mL) to make a homogeneous solution by continuous stirring. These hybrid nanocomposite scaffolds were dipped into the drug-containing beaker and the beaker was wrapped with aluminum foil. These beakers containing corresponding hybrid nanocomposite scaffolds were kept in the oven (50 °C until complete evaporation of solvent) by making small holes in the aluminum foil for slow solvent evaporation to deposit silver sulfadiazine as illustrated in Figure 1C. The silver sulfadiazine release was observed in PBS buffer media under in vitro conditions (pH 7.4 at 37 °C). The specific amount of PBS media (5 mL) was taken from the beaker, and the spectrum was run in a double beam UV–Vis spectrophotometer to record drug release. The PBS buffer solution was taken as a standard reference, and the graphical cumulative release of the drug.

## 3. Characterizations

### 3.1. FTIR Analysis

The FTIR spectrum was recorded to determine the functional group analysis of hybrid nanocomposite scaffolds using Fourier transformation infrared (Shimadzu FTIR-8100A, Tokyo, Japan). The scanning range was 4000–400 cm^−1^ with average scans (150) and resolution 4.0 cm^−1^.

### 3.2. XRD Analysis

The crystalline and amorphous behavior of the hybrid nanocomposite scaffolds was determined using X-ray diffraction (XRD). Bruker AXS D8 (Kontich, Belgium) Advance XRD was used at 40 kV voltage and 30 mA current with Cu Kα radiation (1.540 Å). The XRD analysis was conducted under a fixed angle ranging from 10° to 70°.

### 3.3. SEM-EDS Analysis

The structural morphology of hybrid nanocomposite scaffolds was analyzed using scanning electron microscopy (SEM) (JEOL-JSM 6480, Peabody, MA, USA) coupled with energy dispersive spectroscopy (EDS). These hybrid nanocomposite scaffolds were well-dried and were gold-sputtered before analysis.

### 3.4. Brunauer–Emmett–Teller (BET) Analysis

The Brunauer–Emmett–Teller (Micromeritics Gemini II 2370, Norcross, GA, USA) was used to determine the porosity and pore sizes of hybrid nanocomposite scaffolds. The analysis was performed in triplicate.

### 3.5. Mechanical Testing

The strain–stress analysis of the hybrid nanocomposite scaffolds was measured through a universal testing machine (UTM, Testometrics, Rochdale, UK). The compression strength of the cylindrical hybrid nanocomposite scaffolds (with dimensions 1.5 cm (width) and 1.7 cm (height)) was determined at a speed of 0.5 mm/min. The stress–strain curve was recorded for each sample. The mechanical tests were conducted in triplicate.

### 3.6. Wetting Analysis

The hydrophilic/hydrophobic characteristics of hybrid nanocomposite scaffolds were observed via contact angle meter (XCA-50) (VCA-Optima, AST Inc., Tacoma, WA, USA). The slurry of different formulations was poured into Petri dishes and oven-dried (45 °C) to observe the contact angle. The water drop was placed over dried films after various time intervals (1 and 5 min), and the test was carried out in triplicate.

### 3.7. Swelling Analysis and Biodegradation

The swelling of biomaterials is an important characteristic that facilitates interaction between the biological system and biomaterials. Swelling also regulates the controlled release of therapeutic agents. The well-dried hybrid nanocomposite scaffolds were weighted (50 mg) carefully before analysis. The swelling analysis of hybrid nanocomposite scaffolds was conducted in PBS and deionized water media with a pH of 7.4 at 37 °C. At a specific time, the swollen hybrid nanocomposite scaffolds were taken out by removing excess solvent softly using filter paper to record the actual weight of swelled hybrid nanocomposite scaffolds. These hybrid nanocomposite scaffolds were again put into media to accomplish swelling equilibrium. Equation (1) was used to determine the percentage of swelling.

(1) $$\mathrm{Swelling}\;(\%)\;=\frac{W_{s}{\;-\;W}_{d}}{W_{d}}\;\times \;100$$

where: “$W_{s}$” weight of swollen scaffolds, “$W_{d}$” weight of dry scaffold.

Biodegradation is an essential biomaterial phenomenon that helps quick healing of fractured bone. Each hybrid scaffold was weighed accurately (50 mg) and incubated in the PBS solution with pH 7.4 at 37 °C for 30 days. The scaffold was taken out of excess of PBS solution and rinsed with deionized water. Then it was oven-dried (55 °C) for 1 h. Equation (2) was used to determine the percentage of degradation.

(2) $$\mathrm{Degradation}\;(\%)\;=\frac{W_{i}\;-\;W_{t}}{W_{i}}\;\times \;100$$

where: “*W_t_*” weight of scaffold at time “*t*”, “*W_i_*” initial weight of the scaffold.

### 3.8. Drug Release

The drug-loaded hybrid nanocomposite scaffolds were placed into PBS solution with pH 7.4 at 37 °C. The PBS solution (5 mL) was then taken out after every specific time. The drug release was observed using a double beam UV-Vis spectrophotometer at 297 nm using standard curves. The PBS solution was taken as a reference standard. The cumulative drug release of silver sulfadiazine has been presented via a calibration curve.

### 3.9. In Vitro Biological Activities

#### 3.9.1. Cell Morphology

A cellular morphology study was performed on the hybrid nanocomposite scaffolds (XPH-1, XPH-2, XPH-3, and XPH-4). A fine coating of the scaffold was applied on the bottom of 24-well plates in triplicate. The coated plates were sterilized under UV light for 1 h. MC3T3-E1 cell line (mouse pre-osteoblast cell line) purchased from ATCC was maintained in α-*MEM*, 10% *FBS*, 1% (2 mM) L-glutamine, 1% penicillin/streptomycin media and was seeded (5000 cells per cm^2^) in each well of 24-well plates in triplicate using the same culture media and incubated for 24, 48, and 72 h under standard in vitro conditions (at 37 °C with 5% CO_2_ and 90% humidity). The media were removed manually through a micropipette. Then, absolute ethanol was used to fix the cultured cells (5 min at room temperature). Cell morphologies were investigated under the Nikon ECLIPS TS100 (Melville, NY, USA) fluorescence microscope using a 488 nm excitation filter. Vital dye such as fluorescein diacetate (FDA) was used to avoid the background interference in microscopy created by scaffold coating.

#### 3.9.2. Cell Viability and Optical Density

The cell viability and optical density of these hybrid nanocomposite scaffolds (with concentrations 0.50–2.00 mg/mL) were determined against *MC3T3-E1* cell lines and 0.1% gelatin (+ive control). The cell culture plates were incubated for 24, 48, and 72 h under standard in vitro conditions (37 °C in 5% CO_2_ and 90% humidity). These cells were again incubated into neutral red(NR) media (40 μg/mL) for 2 h using a well-reported method, Repetto et al. [20]. These cells were then washed for 20 min with PBS solution to remove the excess NR stain. Then, dye-staining solution (1% glacial acetic acid, 49% absolute ethanol, and 50% deionized water) was used to destain cells for 10 min. The optical density was observed at 570 nm using an absorbance microplate reader (ELx-800) (BioTek, Winooski, VT, USA). The percentage of the cell viability was calculated with Equation (3).

(3) $$\mathrm{Cell}\;\mathrm{viability}\;(\%)\;=\frac{{OD}_{S}}{{OD}_{C}}\;\times \;100,$$

where: “*OD*_S_” sample concentration, “*OD*_C_” positive control.

### 3.10. Statistical Analysis

The quantitative data were analyzed through statistical analysis system software (IBM SPSS Statistics 21). The data were then presented as mean ± standard error (S.E.) and presented as Y-error bars in figures. The data were performed in triplicate (n = 3), and *p* < 0.05 was considered statistically significant.

## 4. Results and Discussion

### 4.1. FTIR Analysis

**Figure 2:**
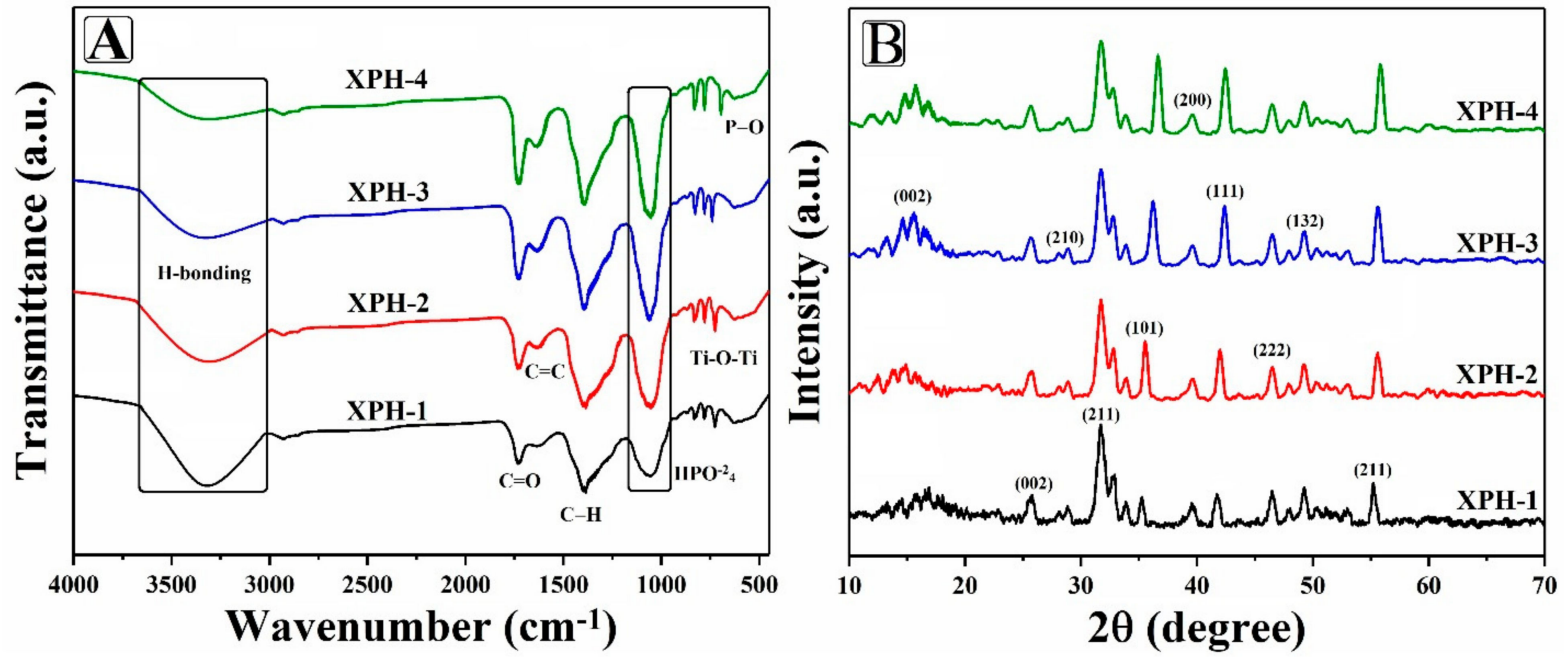
The spectroscopic analysis to determine structural properties of hybrid nanocomposite scaffolds. (**A**) FTIR spectral profiles to analyze the functional groups. (**B**) Diffraction spectra to investigate crystalline and amorphous behavior of the hybrid nanocomposite scaffolds.

Figure 2A presents the FTIR spectra of the fabricated hybrid nanocomposite scaffolds. The spectral profile shows all the characteristic bands of the functional groups of GG-*co*-AAc, HAp NPs, TiO_2_ NPs, and GO. The broadband at 3200–3600 cm^−1^ is attributed to hydrogen bonding (H−bonding). The absorption band at 2927 cm^−1^ is a typical stretching vibration peak of C−H [10]. The absorption bands in the range of 1750–1628 cm^−1^ are attributed to the C−O stretching of GO and N,N’-methylene-bisacrylamide. Since in these hybrid nanocomposite scaffolds, HAp NPs, TiO_2_ NPs, and GO are linked through H−bonding with GG-*co*-AAc polymeric matrix, hence the vibration bands at ~1058 cm^−1^ and ~1300 cm^−1^ are attributed to C–O–H/C deformation modes of polysaccharides, and C–O–H stretching in pyranose of carbohydrates [9]. The absorption peaks at 997, 633, and 543 cm^−1^ represent P–O stretching, O–P–O bending, and HAp NPs, respectively. The characteristic absorption bands at 560–600 cm^−1^ and 845–1000 cm^−1^ are attributed to the calcium and phosphate moiety of HAp NPs. Moreover, the absorption peak at 624 cm^−1^ appears due to −OH, a characteristic peak of HAp NPs [21]. The stretching band from 600–800 cm^−1^ is attributed to TiO_2_ NPs, and it is observed that increasing the amount of TiO_2_ NPs increases the peak intensities [22]. Hence, it is confirmed from FTIR spectral analysis that the hybrid polymeric nanocomposite materials have been successfully fabricated.

### 4.2. XRD Analysis

Figure 2B presents XRD spectral analysis of the TiO_2_/HAp/BG-PVA hybrid nanocomposite scaffolds. The sharp diffraction peaks of HAp NPs were observed at 2θ of 25.15°, 28.80°, 32.10°, 39.80°, 46.50°, and 48.60° with corresponding planes (211), (210), (002), (222), and (132) [10]. The cell parameters for HAp NPs, a = b = 9.4000 and c = 6.9300, were calculated with the standard hexagonal closest packed (HCP) unit cell plane spacing relationship [23]. These corresponded perfectly to the standard data of hydroxyapatite (JCPDS file number 9-0432) [24]. The crystalline size, 23.29 nm, of the HAp NPs was calculated with the Debye–Scherrer equation [25]. The diffraction peaks at 35.10°, 38.21°, 42.21°, and 55.11° corresponding to (101), (200), (111), and (211) were observed for TiO_2_ NPs [26]. The crystal planes parameters were calculated with lattice constants a = b = 3.755 Å and c = 9.5114 Å. These plans may be diffused in hybrid nanocomposite scaffolds and the peak observed at 35.10° (101) confirms TiO_2_ NPs anatase phase in hybrid nanocomposite scaffolds. The diffraction peak of GO has been located at crystalline plane (002) [27]. There was no diffraction peak observed for BG-*co*-AAc due to AX and AA’s amorphous nature that reduces overall crystalline behavior. The reducing crystalline behavior increased the hydrogen bond formation that imprinted HAp NPs/TiO_2_ NPs into the polymeric network.

### 4.3. Morphological and Elemental Analysis

**Figure 3:**
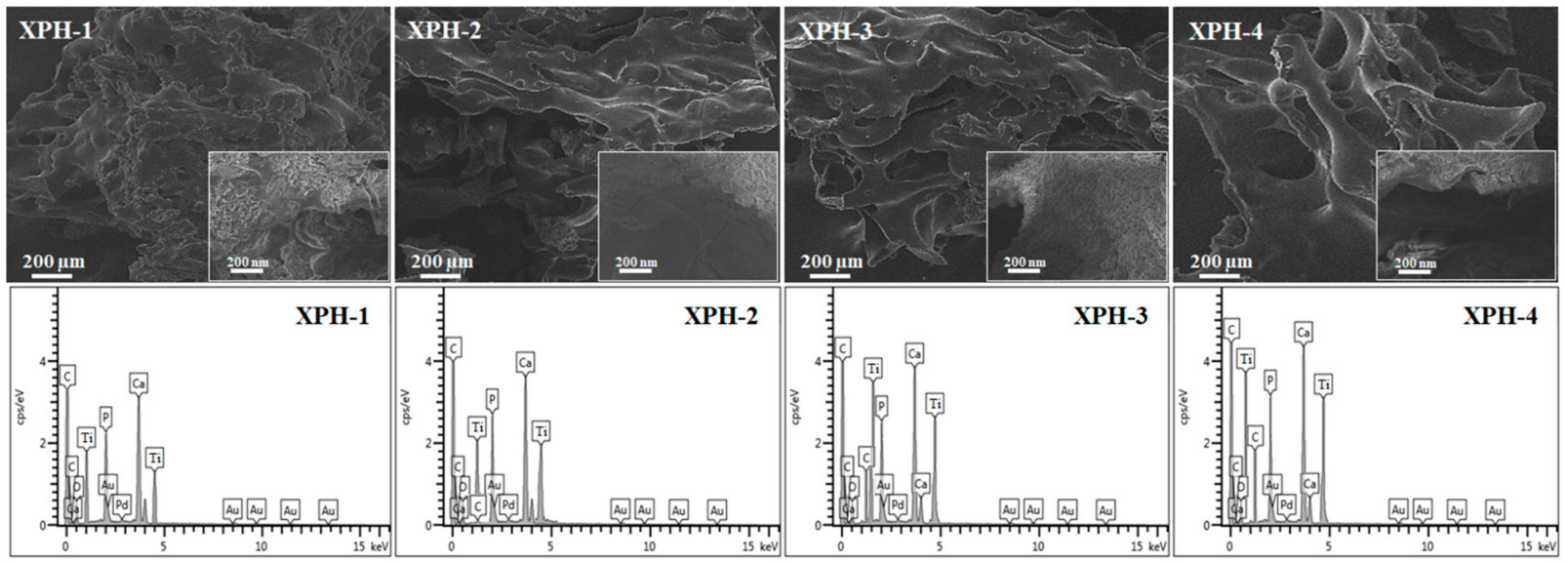
SEM micrographs of the surface of the samples. The inset micrographs show the zoom-in surface of the scaffold samples. The graphs show the EDX spectra of the hybrid nanocomposite scaffolds.

Figure 3 SEM shows the micrographs of the hybrid nanocomposite scaffolds at micro- and nano-scale. All scaffold samples showed evenly distributed connected porosity. The optimum pore size, determined for the scaffold via BET, was 256 μm [28]. This pore size is in the range of the standard pore size required for bone cell growth. The standard pore size for better bone cell infiltration and cell adhesion is approximately 100–350 μm [29]. TiO_2_ NPs played a significant role in controlling pore size and porosity. The increasing amount of TiO_2_ NPs decreased the porosity of these hybrid nanocomposite scaffolds from 79.97 ± 1.32 to 44.32 ± 2.14% and pore size from 256.11 ± 1.28 to 107.42 ± 1.78. This control over the size and porosity of the pores by TiO_2_ NPs is attributed to its ability to crosslink the polymer chains [3,10] physically. The energy dispersive X-ray (EDX) analysis of hybrid nanocomposite scaffolds was performed to determine the chemical composition of hybrid nanocomposite scaffolds. The result shows the existence of carbon (C), calcium (Ca), oxygen (O), phosphorus (P), and titanium (Ti) in the scaffold (Figure 3). The presence of all these elements confirms the existence of TiO_2_/HAp in the polymeric matrix of BG-*g*-AAc [30].

### 4.4. Mechanical Testing and BET Analysis

**Figure 4:**
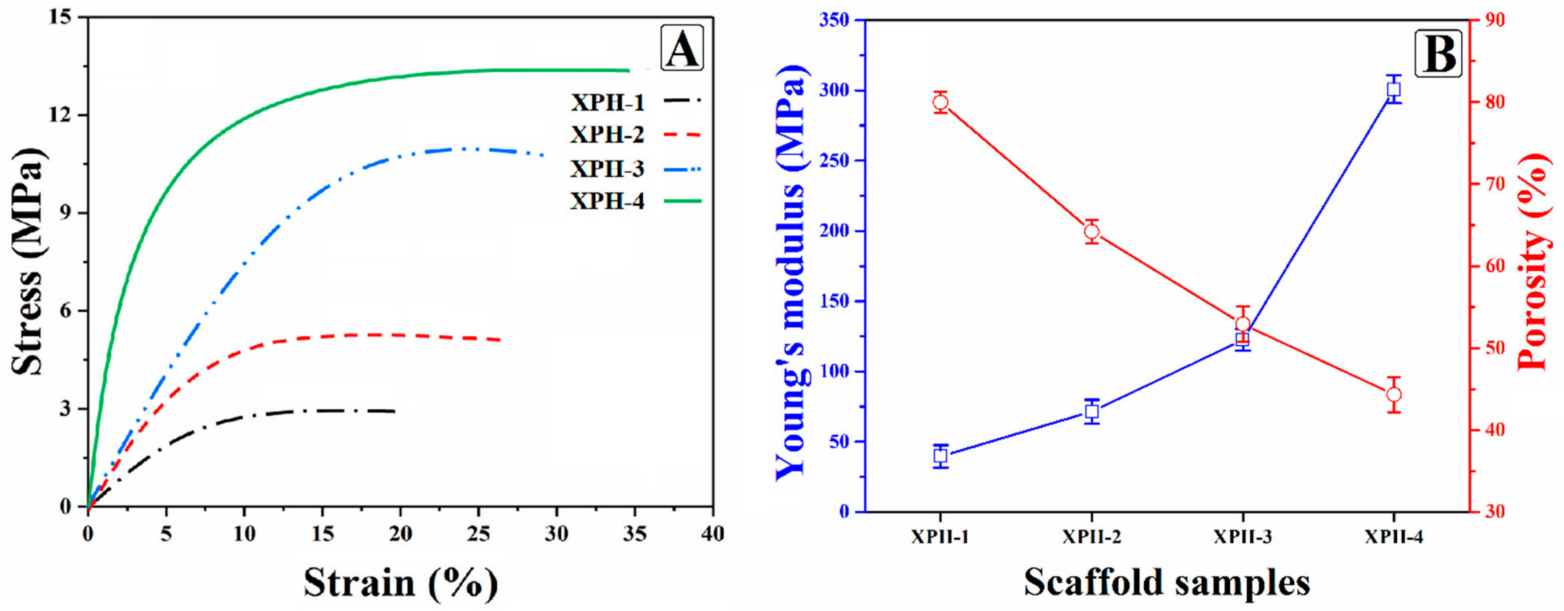
The mechanical behavior of hybrid nanocomposite scaffolds has been investigated through (**A**) stress–strain curve and (**B**) relation of Young’s modulus and porosity.

The mechanical behavior of hybrid nanocomposite scaffolds is presented through the stress–strain curves, as illustrated in Figure 4A. Since GO, HAp NPs, and TiO_2_ NPs are well-known potential materials for structural and enhanced mechanical properties [31,32]. Hence, the optimum amount of GO, HAp NPs, and the increasing amount of TiO_2_ increases the compression strength from 2.96 ± 1.34 to 13.31 ± 2.45 MPa and 19.77 ± 1.50 to 34.89 ± 1.56% with a maximum Young’s modulus 300.81 ± 1.90 MPa as details have been mentioned in Table 1. It is also observed that the increasing amount of TiO_2_ decreases the porosity (79.97 ± 1.32 to 44.32 ± 2.14%) and pore size (256.11 ± 1.28 to 107.42 ± 1.78 μm). The mechanical and porosity data of hybrid nanocomposite scaffolds are presented in Table 1. The increasing amount of TiO_2_ NPs provides the additional active sites, which act as crosslinking that increases the mechanical properties of the hybrid nanocomposite scaffolds by reducing porosity and pore size [33]. The relationship between mechanical behavior and porous properties is presented in Figure 4B. The different amount of TiO_2_ NPs alters the chemical structures that affect interface and matrix grains. HAp NPs play a vital role in mechanical and structural mechanical strength by improving mechanical strength because of grain size and grain boundary. The nanosize material provides a higher surface area that interacts with other materials to enhance mechanical strength by regulating porosity and pore size [34].

### 4.5. Swelling, Biodegradation, and Wetting Analysis

**Figure 5:**
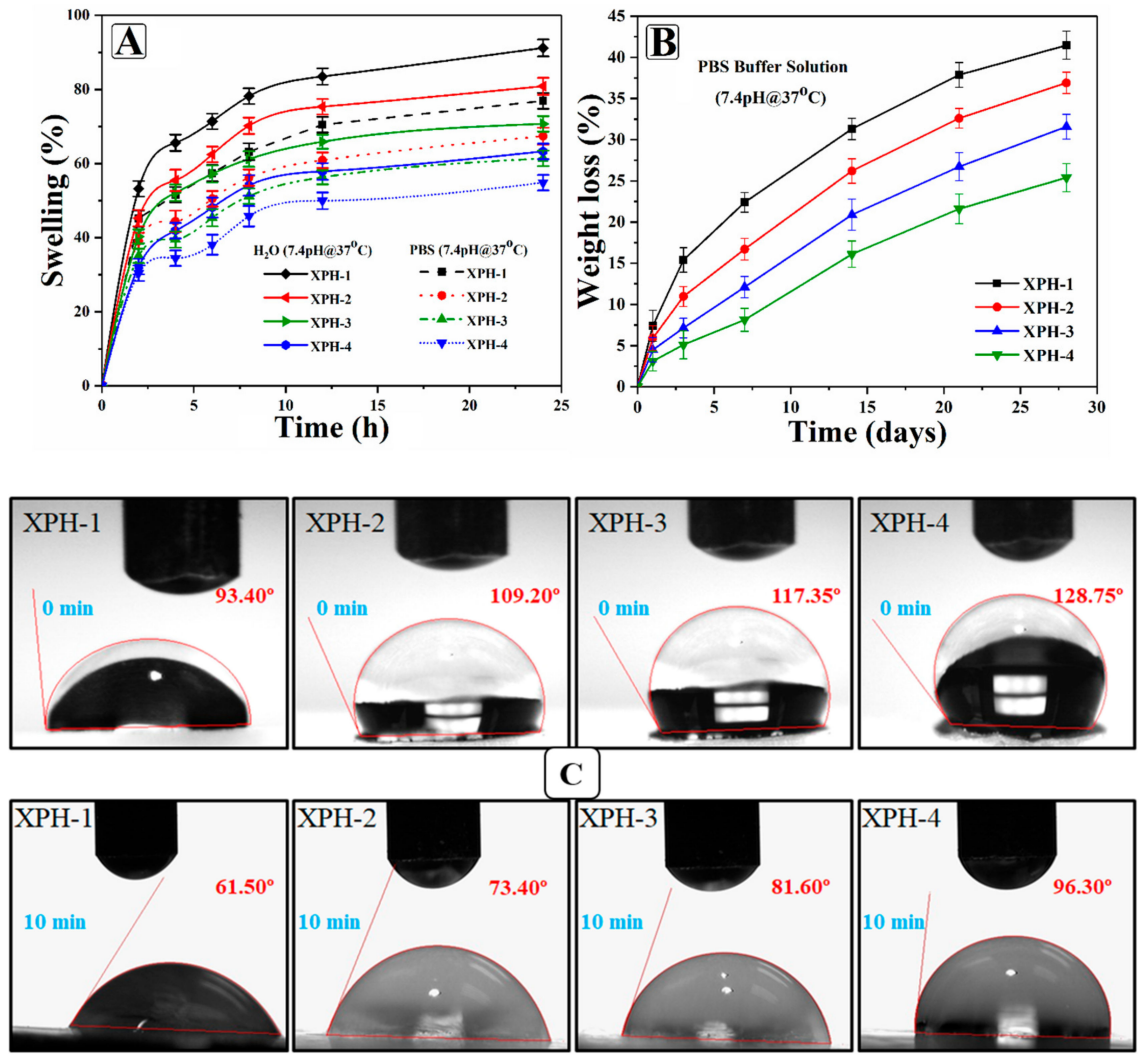
(**A**) Swelling analysis of the hybrid nanocomposite scaffolds in different media (pH 7.4) at 37 °C, (**B**) degradation of hybrid nanocomposite scaffolds samples in PBS solution (pH 7.4) at 37 °C, and (**C**) wetting analysis of scaffold samples to determine hydrophilicity and hydrophobicity at room temperature.

When the biomaterial is in contact with the body, it exhibits different behavior and properties during that interaction. It starts absorbing fluids and starts swelling, and after a particular period, it starts degrading to reproduce or regenerate new tissues. Hence, the importance of wet polymeric matrix of hybrid nanocomposite scaffolds cannot be denied for cell adherence, proliferation, and migration in tissue engineering to repair defected organs [35]. These fabricated hybrid nanocomposite scaffolds have different swelling behavior due to the different amount of TiO_2_ NPs. Figure 5A shows the swelling kinetics of the hybrid nanocomposite scaffolds in deionized water and reduced swelling in PBS solution. It is evident from the data that increasing the amount of TiO_2_ NP decreased the swelling of the hybrid nanocomposite scaffolds both in deionized water and PBS media at 37 °C. XPH-1 exhibited maximum swelling (deionized water = 91.20%, PBS media = 76.90%) due to least amount of TiO_2_ NP. On the other hand, XPH-4 had minimum swelling (deionized water = 63.30%, PBS media = 54.87%) since this sample had the maximum amount of TiO_2_ NP content. The varying swelling characteristics of these hybrid nanocomposite scaffolds can be attributed to the very interesting phenomenon where TiO_2_ NP acts as a physical crosslinker [36]. The increasing amount of TiO_2_ NPs may generate additional crosslinking within polymeric material that decreases material elastic behavior [37].

The degradation of hybrid nanocomposite scaffolds was investigated in PBS solution at 37 °C, and the percentage of degradation was determined by weight loss as presented in Figure 5B. The increased weight loss was observed as the immersion time in the PBS solution increased. The increasing amount of TiO_2_ NPs has an inverse effect on degradation since these are physical crosslinkers. The scaffold sample XPH-1 exhibited maximum degradation and XPH-4 minimum degradation. The different degradation properties of hybrid nanocomposite scaffolds are attributed to the crosslinking ability of the TiO_2_ NPs. It would be convenient to conclude that increasing the amount of TiO_2_ NPs results in increased crosslinking of the hybrid nanocomposite scaffolds, which leads to a more compact structure that is not easily eroded. The increased crosslinking also shifts the already hydrophobic nature of the scaffold to a relatively more hydrophobic nature (Figure 5C) [38].

The wetting analysis of hybrid nanocomposite scaffolds has been analyzed via water contact angle (WCA) measurement to analyze the hydrophilic and hydrophobic behavior of the scaffold. The hydrophilic and hydrophobicity boundary of WCA is 90° [39,40]. Figure 5C shows the WCA for all the fabricated hybrid nanocomposite scaffolds. The WCA of the scaffold increases from 93° (XPH-1) to 120° (XPH-4) with the increase in the amount of TiO_2_ NPs. It shows that the hydrophobic character of the scaffold increases with an increase in the TiO_2_ NPs. This is well attributed to the TiO_2_ NPs ability to crosslink the scaffold physically.

### 4.6. Release of Silver Sulfadiazine

**Figure 6:**
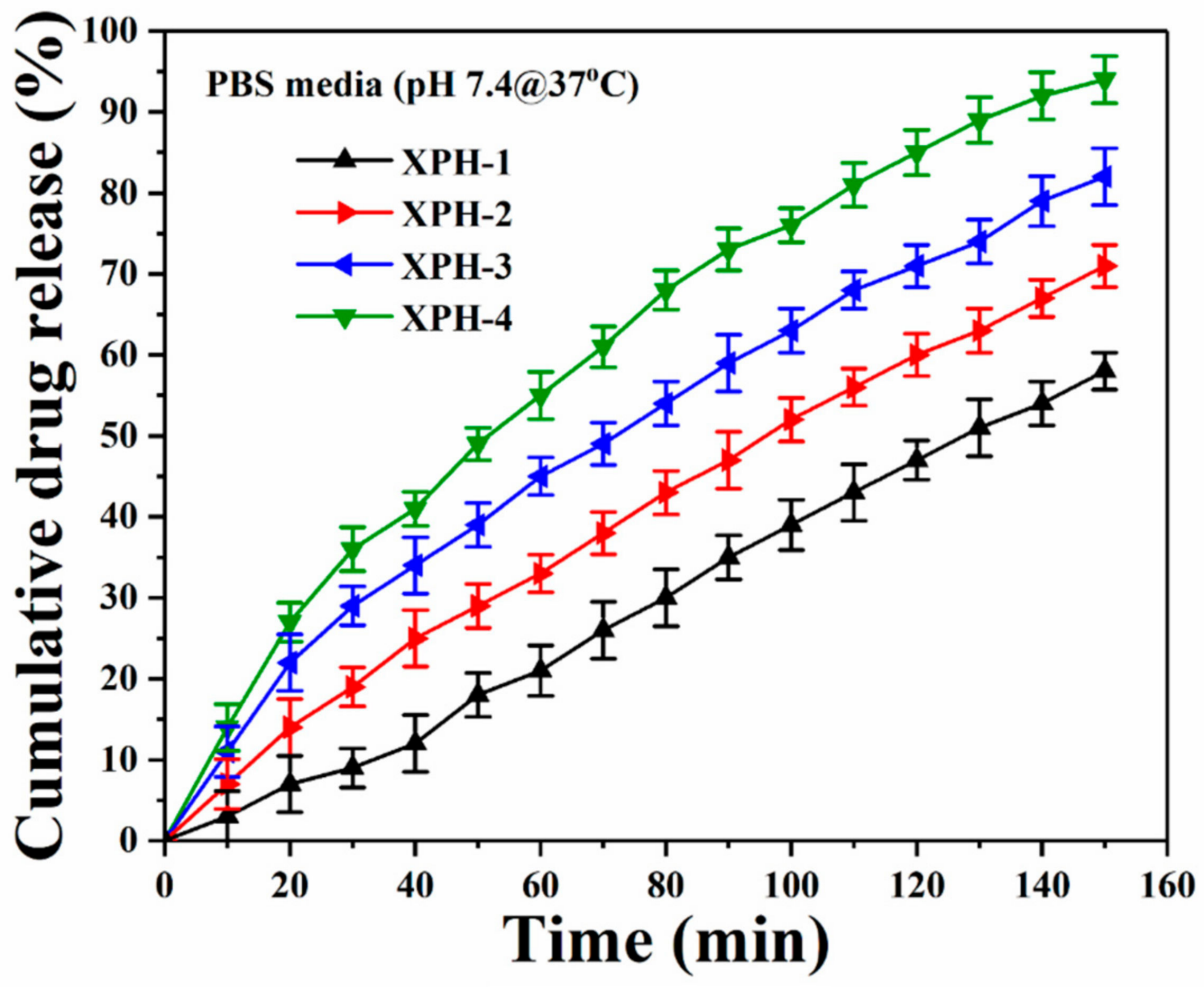
The drug release profile of all polymeric hybrid nanocomposite scaffolds in PBS solution at 37 °C.

The PBS solution was used to determine silver sulfadiazine release at 37 °C to deal with pathogenic activities at the fracture site during implantation. The sustained release of silver sulfadiazine is shown in Figure 6. The sustained, gradual, and prolonged antibacterial drug is an essential phenomenon for antibacterial activities [41]. It was seen from the drug release profile that initially all samples showed quick release but sustained drug release became continuous after 3 h. Sample XPH-4 has shown maximum drug release as ~14.5% after 10 min and ~94.1% after 150 min.

In contrast, the least drug release was observed for Sample XPH-1, i.e., ~3.7% after 10 min and ~58.2% after 60 min. After 6 h, continuous but sustained release of drug was observed. However, controlled drug release was observed from XPH-4 to XPH-1 due to the different structural properties of the polymeric hybrid nanocomposite scaffolds. Since XPH-4 had the maximum amount of TiO_2_ NPs and possessed maximum active site but less H-bonding, the quick release of the drug was observed. On the other hand, XPH-1 had the least amount of GO and had less charge density. It had less capability to attach drugs through H-bonding [42]. The drug may interact more with the polymeric matrix via van der Waal or other attraction forces. That is why sustained drug release was found for sample XPH-1; maximum drug was to show drug release. Hence, sustained and controlled release of the drug is vital to kill disease-causing bacteria during and after bone implantation.

### 4.7. In Vitro Activities

#### 4.7.1. Cell Viability and Optical Density

**Figure 7:**
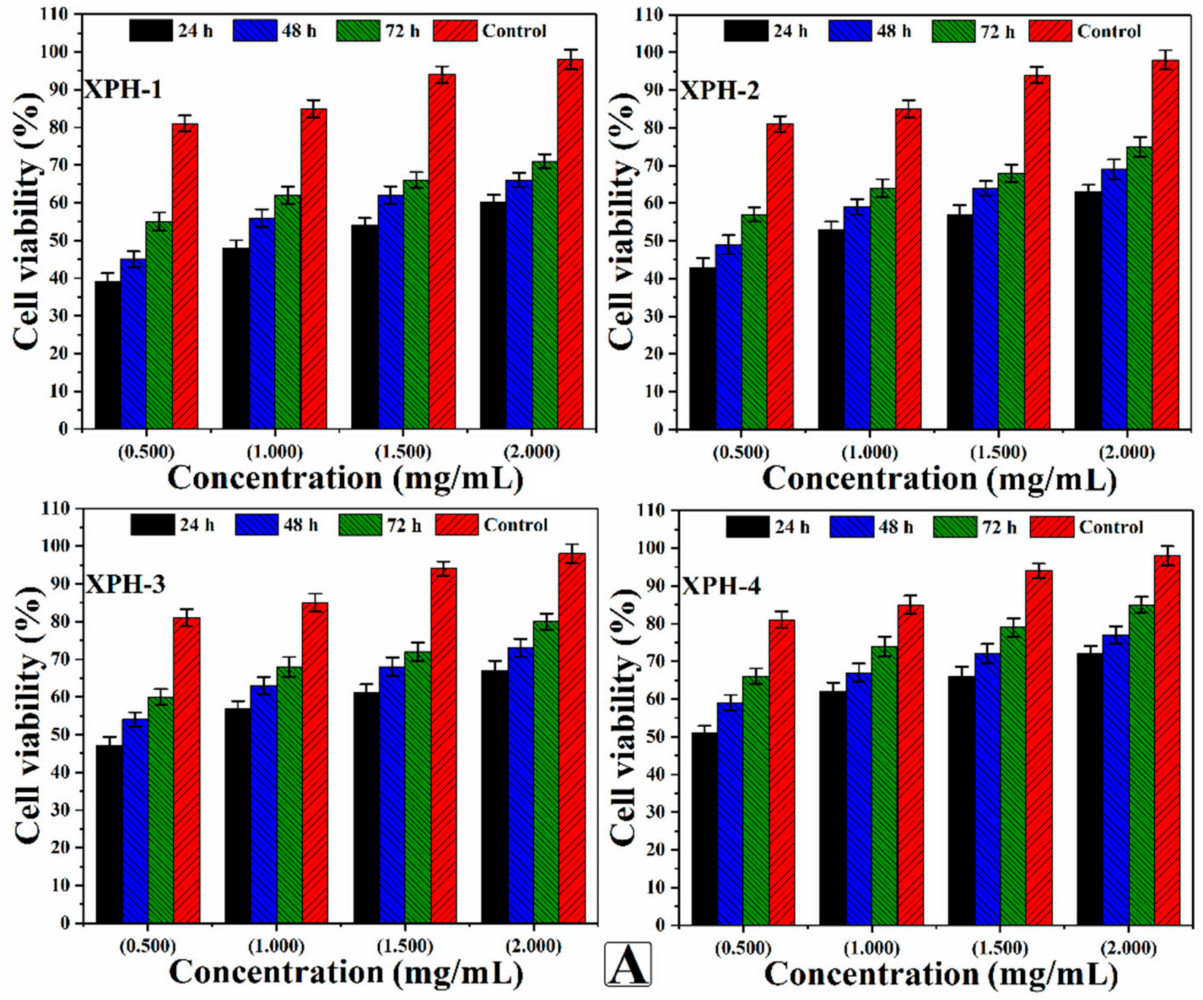

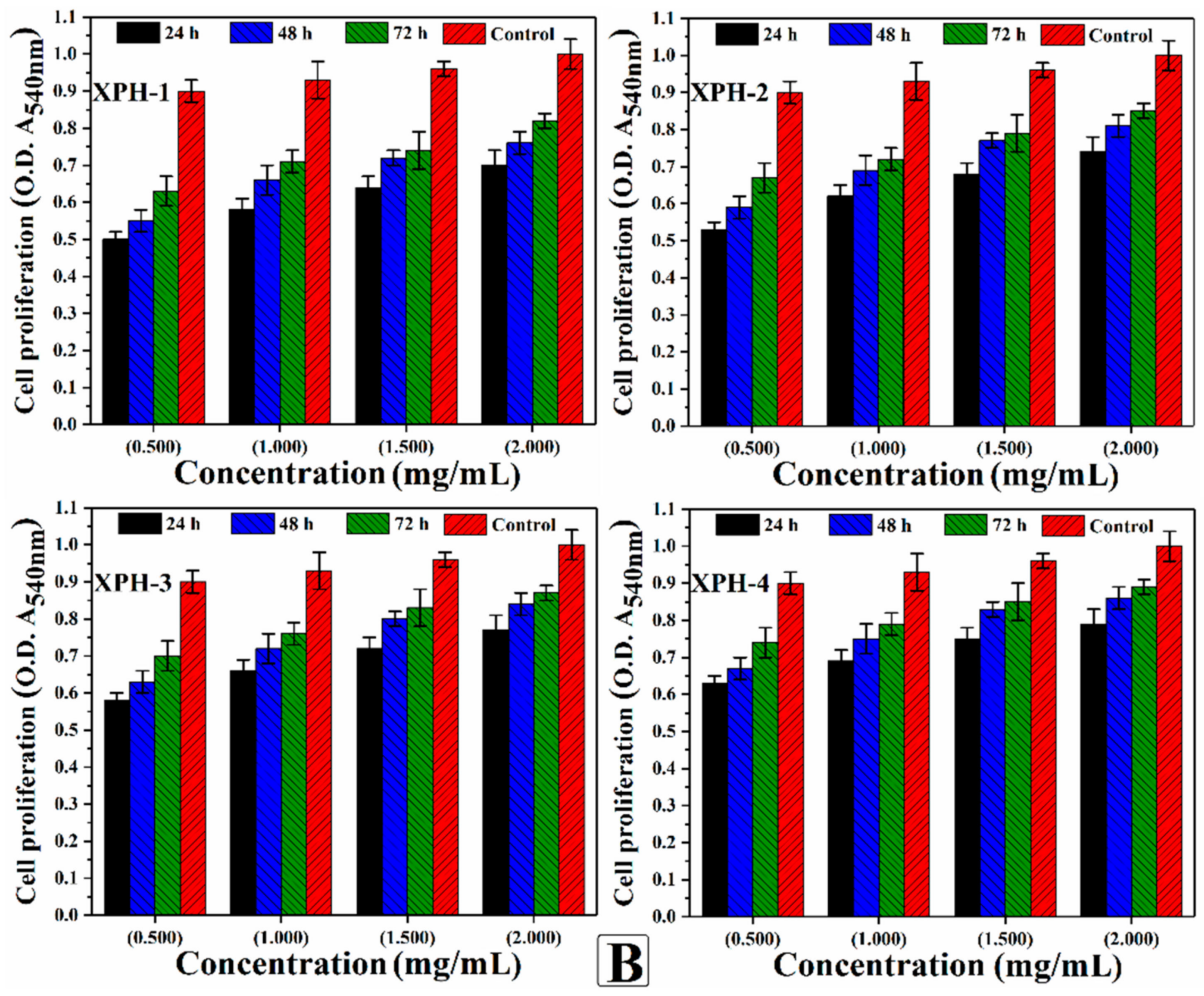
Presents the cellular behavior of *MC3T3-E1* against different concentrations (0.500, 1.000, 1.500, and 2.000 mg/mL) of polymeric hybrid nanocomposite scaffolds at different time intervals (24, 48, and 72 h) to (**A**) determine cell viability and (**B**) optical density under standard in vitro conditions.

In vitro study of all scaffold specimens against MC3T3-E1 cell lines was performed to determine the bioactivities (i.e., cell viability assay and optical density). The cell viability (Figure 7A) and optical density (Figure 7B) were performed against different concentrations (0.500, 1.000, 1.500, and 2.000 mg/mL) to investigate the effect of concentration. Scaffold samples were incubated under necessary in vitro conditions with *MC3T3-E1* cell lines. The cell viability and optical density were recorded after a different time interval (24, 48, and 72 h) [43]. It was observed that increasing concentration and time caused increasing cell viability and cell proliferation. Hence, maximum cell viability and cell proliferation values were observed at maximum concentration and after 72 h for XPH-4. The scaffold sample XPH-1 contained the maximum amount of TiO_2_ NPs_,_ which offer an extra active site that helps cell adherence, which leads to cell proliferation with cell cytotoxicity [44]. Meanwhile, GO has several oxygen-based functional groups, and it not only enhances microstructural properties, but also facilitates cell adherence and cell proliferation. Whereas hydroxyapatite is famous for enhancing the osteogenesis process. The combined effect of TiO_2_, HAp NPs, and GO alters the physicochemical characteristics that support cellular compatibility. Hence, these scaffold samples have presented cytocompatibility with enhanced cell viability and proliferation, which confirms the biocompatible nature of fabricated hybrid nanocomposite scaffolds.

#### 4.7.2. Cell Morphology

**Figure 8:**
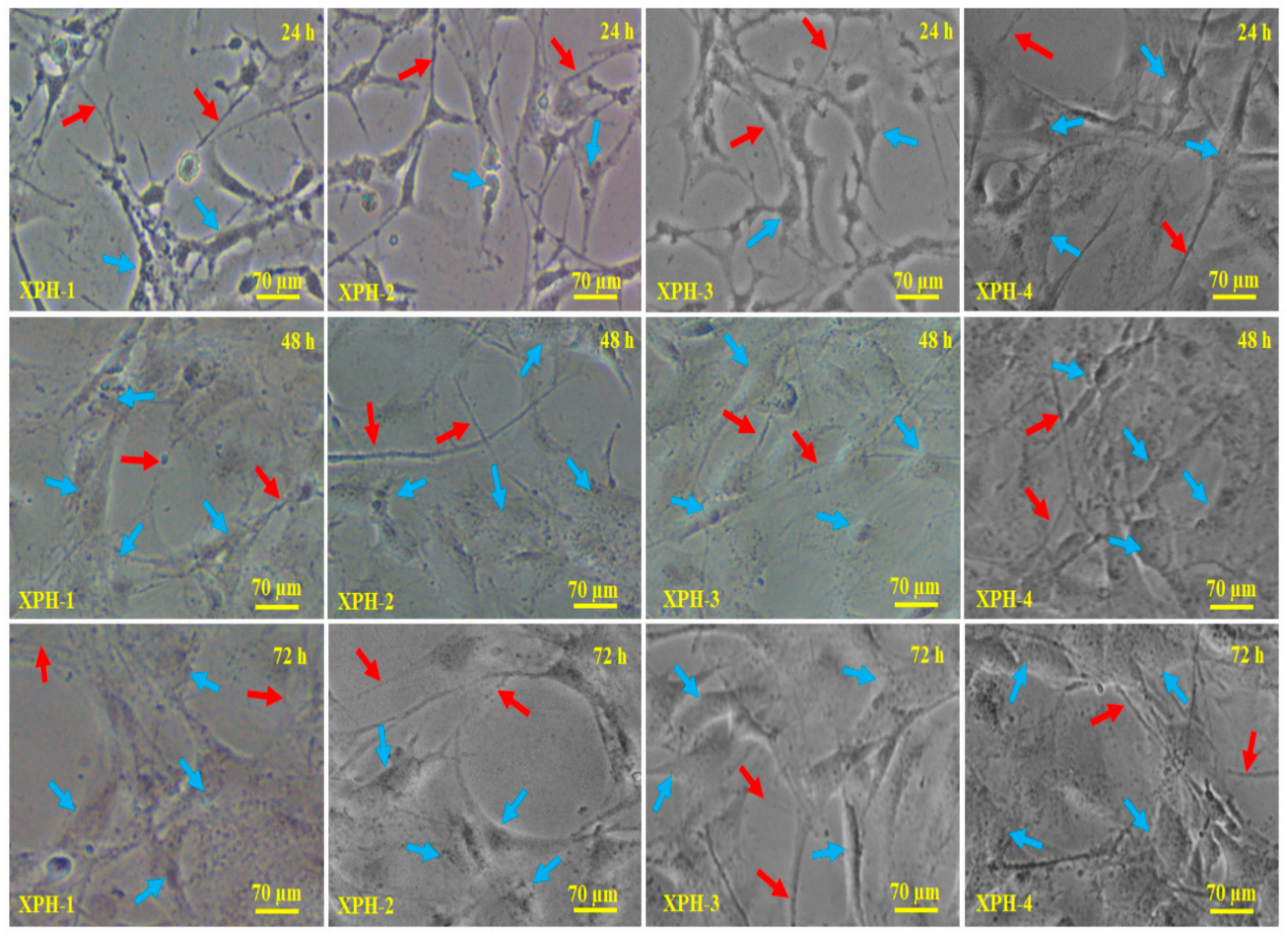
In vitro cell morphology of *MC3T3-E1* cells onto the hybrid nanocomposite scaffolds (XPH-1, XPH-2, XPH-3, and XPH-4) at different times (24, 48, and 72 h). The red arrows present the thread-like morphologies of the cells whereas the blue arrows show the mature cylinder-like morphologies of the cell.

The scaffold samples were treated with *MC3T3-E1* to determine cell adherence and cell morphology, as presented in Figure 8. These scaffold samples are multifunctional (–COOH, –OPO_3_, –H, and –OH, etc.) due to GO, HAp NPs, TiO_2_ NPs, and polymeric matrix and these factors all together facilitate cell adherence and proliferation with a proper cylindrical shape. These functionalities establish hydrogen bonding with the cell membrane [45]. The increasing amount of TiO_2_ NPs increases the active site, which encourages cell adherence, differentiation, and growth due to integrin bonding with the material surface. Initially, the shape of the cell was like a thread (red arrows), and after 24 h, more cell adherence was observed (blue arrow). The scaffold samples (XPH-3 and XPH-4) have a microstructural surface with rough morphology, and after increasing time, clearer cell spreading was observed. Hence, a considerable change in absorbance was presented at different time intervals. The wetting behavior also facilitated communication of material with DNA of host bone that encouraged fracture cell adherence and cell growth to form new bone to regenerate bone [46].

Furthermore, after 48 h, more cell adherence was observed after quick cylindrical morphology and cell proliferation for all samples. Still, a substantial difference was observed, especially for XPH-3 and XPH-4, as shown in Figure 8. Due to the increased amount of TiO_2_ NPs with optimized GO, it induced their functionalities throughout the scaffold materials by enhancing physicochemical and biomechanical characteristics. It can also be explained that increasing the incubation time makes the surface more hydrophilic, thus encouraging more cell adherence through hydrogen bonding. Hence, the multifunctional biomaterial is necessary for cell growth to heal fracture bone [47].

#### 4.7.3. SEM Analysis of Cell Culture and Attachment

**Figure 9:**
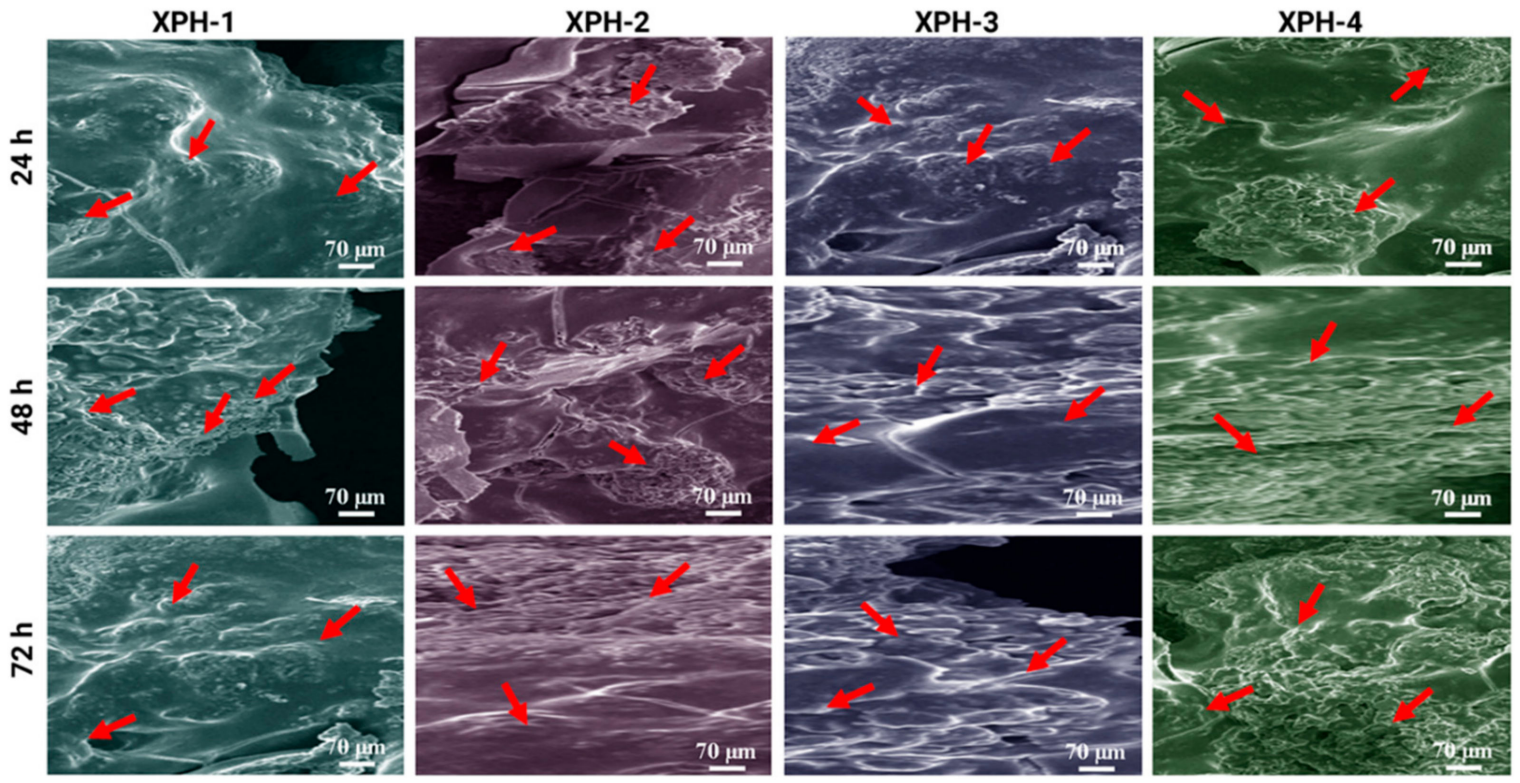
Presents the SEM analysis of cell culture and adherence for all hybrid nanocomposite scaffolds against *MC3T3-E1* after 24, 48, and 72 h and red arrows indicate the culture and adherence of osteoblast cells.

The cell adherence and morphology of *MC3T3-E1* over the surface of the scaffold have been observed through SEM after 72 h of cell culture (Figure 9). Cell adherence increased as the amount of TiO_2_ NPs, increased from XPH-1 to XPH-4. The synergic effect of the TiO_2_ NPs and GO led to the creation of additional active sites. Cell adherence over the scaffold is visible with different shapes. Most of the cells exhibit well-spreading cylindrical shapes (as evident in the cell morphological section) on hybrid nanocomposite scaffolds by covering the surface [48]. The cell penetration into pore and growth over the surface increased as time increased, and cell culturing is presented with red arrows after 24, 48, and 72 h, as shown in Figure 9. These increasing multifunctional properties and active sites facilitate cell adherence. It also may be explained based on wetting phenomena that hydrogen bonding encourages cell adherence. Hence, rough surface morphologies with enhanced multifunctional properties and wetting together encourage cell adherence due to increasing oxygen-based functional groups that offer H-bonding [49]. The increasing time also improves cell adherence and proliferation by gene expression that facilitates new bone growth to heal fractured bone.

## 5. Conclusions

The polymeric nanocomposites were synthesized through free radical polymerization to fabricate porous scaffolds via the freeze-drying technique. The physicochemical (morphology, porosity, wetting, swelling, biodegradation, and biomechanical, etc.) properties of the hybrid nanocomposite scaffolds were performed using different techniques. During analysis, it was observed that these properties can be optimized efferently by varying amounts of TiO_2_ NPs. The addition of optimized GO and different amounts of TiO_2_ NPs into the polymeric matrix enhanced the physicochemical and biomechanical characteristics of fabricated hybrid nanocomposite scaffolds that encouraged proliferation and cell attachment against *MC3T3-E1* cell lines. XPH-4 exhibited the maximum mechanical behavior (strength = 13.31 ± 2.45 MPa, Young’s modulus = 300.85 ± 1.90 MPa) with less pore size (107.42 ± 1.78 μm) and porosity (44.32 ± 2.14%) than other scaffold samples and XPH-1 had the least mechanical properties (strength = 2.96 ± 2.14 MPa, Young’s modulus = 39.56 ± 2.14 MPa) with maximum pore size (256.11 ± 1.28 μm) and porosity (79.97 ± 1.32%) with ~94.1% after 150 min. Whereas, XPH-4 had the best biocompatibility, cell adherence, and proliferation with uniform interrelated porous structure and load-bearing behavior among all other scaffold samples. Hence, it is concluded from the consistent results of our studies that these hybrid nanocomposite scaffolds would be potential biomaterials in bone tissue engineering. Therefore, these hybrid nanocomposite scaffolds can be implanted to treat different bones with different mechanical and morphological properties to engineer the fracture bone tissue.

## Figures and Tables

**Table 1:** The mechanical properties, molar crosslink density, pore size, and porosity of polymeric hybrid nanocomposite scaffolds.

Sample	Strain (%)	Strength (MPa)	Young’s Modulus (MPa)	Molar Crosslink Density (mol/m^3^)	Pore Size (μm)	Porosity (%)
**XPH-1**	19.77 ± 1.50	2.96 ± 1.34	39.56 ± 2.14	0.0053	256.11 ± 1.28	79.97 ± 1.32
**XPH-2**	26.64 ± 2.36	5.18 ± 2.60	71.43 ± 1.36	0.0095	195.64 ± 1.64	64.23 ± 1.43
**XPH-3**	29.53 ± 1.90	10.68 ± 1.12	122.45 ± 2.24	0.0164	142.17 ± 2.47	52.91 ± 2.17
**XPH-4**	34.89 ± 1.56	13.31 ± 2.45	300.85 ± 1.90	0.0402	107.42 ± 1.78	44.32 ± 2.14
